# 2342. Evaluation of mRNA-LNP and adjuvanted protein SARS-CoV-2 vaccines in a maternal antibody mouse model

**DOI:** 10.1093/ofid/ofad500.1964

**Published:** 2023-11-27

**Authors:** Ross N England, Elizabeth Drapeau, Reihaneh Hosseinzadeh, Drew Weissman, Scott Hensley

**Affiliations:** Children's Hospital of Philadelphia, Havertown, Pennsylvania; University of Pennsylvania, Philadelphia, Pennsylvania; Acuitas Therapeutics, Burnaby, British Columbia, Canada, Pennsylvania; University of Pennsylvania, Philadelphia, Pennsylvania; University of Pennsylvania, Philadelphia, Pennsylvania

## Abstract

**Background:**

Maternal antibodies (matAbs) protect against a myriad of pathogens early in life; however, these antibodies can also inhibit *de novo* immune responses against some vaccine platforms. Severe acute respiratory syndrome coronavirus 2 (SARS-CoV-2) matAbs are efficiently transferred during pregnancy and protect infants against subsequent SARS-CoV-2 infections. It is unknown if matAbs inhibit immune responses elicited by different types of SARS-CoV-2 vaccines.

**Methods:**

We established a mouse model to determine if SARS-CoV-2 spike (S)-specific matAbs inhibit immune responses elicited by recombinant protein and nucleoside-modified mRNA-lipid nanoparticle (mRNA-LNP) vaccines. Mouse dams were vaccinated with SARS-CoV-2 S protein-encoding mRNA-LNP vaccine (or vehicle) in early pregnancy and pups were born and matAbs quantitated in pup serum by ELISA. Pups with and without matAbs were vaccinated at weaning with recombinant S protein (adjuvanted with Addavax or empty LNP) or mRNA-LNP and serum S-specific IgG quantitated over time.

**Results:**

S-specific matAbs were transferred to pups and decayed over time as expected. We found that SARS-CoV-2 mRNA-LNP vaccines elicited robust *de novo* antibody responses in mouse pups in the presence of matAbs. Recombinant protein vaccines were also able to circumvent the inhibitory effects of matAbs when co-administered with Addavax or empty LNP as adjuvants.

**Conclusion:**

While additional studies need to be completed in humans, our results raise the possibility that mRNA-LNP-based and adjuvanted protein-based SARS-CoV-2 vaccines have the potential to be effective when delivered in the presence of matAbs.

**Disclosures:**

**Reihaneh Hosseinzadeh, MASc**, Acuitas Therapeutics: Employee **Drew Weissman, MD, PhD**, Intellectual Property/Patents: D.W. is named on the first patent describing nucleoside-modified mRNA and on patents describing its use as a treatment and vaccine platform. **Scott Hensley, PhD**, Intellectual Property/Patents: SEH is named on patents that describe the use of nucleoside-modified mRNA as a platform to deliver therapeutic proteins and as a vaccine platform

